# Impact of advance directives on the variability between intensivists in the decisions to forgo life-sustaining treatment

**DOI:** 10.1186/s13054-020-03402-7

**Published:** 2020-12-02

**Authors:** Margot Smirdec, Mercé Jourdain, Virginie Guastella, Céline Lambert, Jean-Christophe Richard, Laurent Argaud, Samir Jaber, Kada Klouche, Anne Medard, Jean Reignier, Jean-Philippe Rigaud, Jean-Marc Doise, Russell Chabanne, Bertrand Souweine, Jeremy Bourenne, Julie Delmas, Pierre-Marie Bertrand, Philippe Verdier, Jean-Pierre Quenot, Cecile Aubron, Nathanael Eisenmann, Pierre Asfar, Alexandre Fratani, Jean Dellamonica, Nicolas Terzi, Jean-Michel Constantin, Axelle Van Lander, Renaud Guerin, Bruno Pereira, Alexandre Lautrette

**Affiliations:** 1 Department of Anaesthesiology and Critical Care Medicine, Estaing Hospital, University Hospital of Clermont-Ferrand, Clermont-Ferrand, France; 2 INSERM U1190, CHU Lille, Department of Critical Care Medicine, Roger Salengro Hospital, Univ. Lille, 59000 Lille, France; 3 Palliative Care Unit, Louise Michel Hospital, University Hospital of Clermont-Ferrand, Clermont-Ferrand, France; 4 Biostatistics Unit (DRCI), University Hospital of Clermont-Ferrand, Clermont-Ferrand, France; 5 Medical Intensive Care Unit, La Croix Rousse Hospital, University Hospital of Lyon, Lyon, France; 6 Medical Intensive Care Unit, Edouard Herriot Hospital, University Hospital of Lyon, Lyon, France; 7 Department of Anaesthesiology and Critical Care Medicine, Saint Eloi Hospital, University Hospital of Montpellier, Montpellier, France; 8 Medical Intensive Care Unit, Lapeyronnie Hospital, University Hospital of Montpellier, Montpellier, France; 9 Cardiac Surgery Intensive Care Unit, Department of Anaesthesiology and Critical Care Medicine, Montpied Hospital, University Hospital of Clermont-Ferrand, Clermont-Ferrand, France; 10 Medical Intensive Care Unit, Hotel-Dieu Hospital, University Hospital of Nantes, Nantes, France; 11 Intensive Care Unit, Pasteur Hospital, Hospital of Dieppe, Dieppe, France; 12 Intensive Care Unit, Morey Hospital, Hospital of Chalon-Sur-Saône, Chalon-sur-Saône, France; 13 Neurocritical Care Unit, Department of Anaesthesiology and Critical Care Medicine, Montpied Hospital, University Hospital of Clermont-Ferrand, Clermont-Ferrand, France; 14 Medical Intensive Care Unit, Montpied Hospital, University Hospital of Clermont-Ferrand, Clermont-Ferrand, France; 15 Emergency Intensive Care Unit, La Timone Hospital, University Hospital of Marseille, Marseille, France; 16 Intensive Care Unit, Puel Hospital, Hospital of Rodez, Rodez, France; 17 Intensive Care Unit, Veil Hospital, Hospital of Cannes, Cannes, France; 18 Intensive Care Unit, Hospital of Montluçon, Montluçon, France; 19 Medical Intensive Care Unit, Mitterrand Hospital, University Hospital of Dijon, Dijon, France; 20 Medical Intensive Care Unit, Centre Hospitalier Universitaire de Brest, Université de Bretagne Occidentale, Brest, France; 21 Intensive Care Unit, Centre Jean Perrin, 54 Rue Montalembert, BP69, 63003 Clermont-Ferrand, Cedex 1, France; 22 Medical Intensive Care Unit, Larrey Hospital, University Hospital of Angers, Angers, France; 23 Intensive Care Unit, Department of Anaesthesiology and Critical Care Medicine, Saint-Louis Hospital, Assistance Publique Hopitaux de Paris, Paris, France; 24 Medical Intensive Care Unit, l’Archet Hospital, University Hospital of Nice, Nice, France; 25 Medical Intensive Care Unit, Michallon Hospital, University Hospital of Grenoble, Grenoble, France; 26 GRC 29, AP-HP, DMU DREAM, Department of Anaesthesiology and Critical Care, Pitié-Salpêtrière Hospital, Sorbonne University, Paris, France; 27 UPU ACCePPt, Université Clermont Auvergne, Clermont-Ferrand, France; 28 EA-481, Laboratoire de Neurosciences, UBFC, Besançon, France; 29 Intensive Care Unit, Department of Anaesthesiology and Critical Care Medicine, Estaing Hospital, University Hospital of Clermont-Ferrand, Clermont-Ferrand, France; 30 LMGE «Laboratoire Micro-Organismes: Génome Et Environnement», UMR CNRS 6023, Clermont-Auvergne University, Clermont-Ferrand, France; 31 Intensive Care Medicine, Montpied Teaching Hospital, 54 Rue Montalembert, BP69, 63003 Clermont-Ferrand, Cedex 1, France

**Keywords:** Advance directives, Decisions to forgo life-sustaining treatment, ICU

## Abstract

**Background:**

There is wide variability between intensivists in the decisions to forgo life-sustaining treatment (DFLST). Advance directives (ADs) allow patients to communicate their end-of-life wishes to physicians. We assessed whether ADs reduced variability in DFLSTs between intensivists.

**Methods:**

We conducted a multicenter, prospective, simulation study. Eight patients expressed their wishes in ADs after being informed about DFLSTs by an intensivist-investigator. The participating intensivists answered ten questions about the DFLSTs of each patient in two scenarios, referring to patients’ characteristics without ADs (round 1) and then with (round 2). DFLST score ranged from 0 (no-DFLST) to 10 (DFLST for all questions). The main outcome was variability in DFLSTs between intensivists, expressed as relative standard deviation (RSD).

**Results:**

A total of 19,680 decisions made by 123 intensivists from 27 ICUs were analyzed. The DFLST score was higher with ADs than without (6.02 95% CI [5.85; 6.19] vs 4.92 95% CI [4.75; 5.10], *p* < 0.001). High inter-intensivist variability did not change with ADs (RSD: 0.56 (round 1) vs 0.46 (round 2), *p* = 0.84). Inter-intensivist agreement on DFLSTs was weak with ADs (intra-class correlation coefficient: 0.28). No factor associated with DFLSTs was identified. A qualitative analysis of ADs showed focus on end-of-life wills, unwanted things and fear of pain.

**Conclusions:**

ADs increased the DFLST rate but did not reduce variability between the intensivists. In the decision-making process using ADs, the intensivist’s decision took priority. Further research is needed to improve the matching of the physicians’ decision with the patient’s wishes.

*Trial registration* ClinicalTrials.gov Identifier: NCT03013530. Registered 6 January 2017; https://clinicaltrials.gov/ct2/show/NCT03013530.

## Background

A decision to forgo life-sustaining treatment (DFLST) is made by 3–30% of intensive care unit (ICU) patients and is recorded in 90% of decedent patients [1]. The DFLST includes decisions about no-escalation or withholding or withdrawal of treatment that lead to differences in mortality [2]. These decisions are made by the patient, the physician or close relatives, or result from a shared decision-making process. However, there are numerous limitations to this process. A DFLST made by the family can be influenced by their preferences [3] or by the psychological symptoms associated with ICU admission such as anxiety/depression and post-traumatic stress disorder, which prevent the patient’s wishes being clearly reported [4]. In addition, misperceptions about the patient’s prognosis by the surrogate can lead to differing expectations by physicians and family and delay decision-making [5, 6]. When making a DFLST, physicians are greatly influenced by their personal characteristics including religion and culture [7, 8], which results in considerable variability in their decisions [7, 9]. This variability is constant within the same specialty or structure [10–14]. Patients want physicians to follow their wishes [15], but most ICU patients are not able to properly communicate these wishes because they lack decision-making capacity. Advance directives (ADs) give incapacitated patients the opportunity to indicate what treatment they wish to have [16]. There is a worldwide consensus that physicians should respect the patient’s ADs [17]. In a given setting, when the respect of the patient’s wishes has priority over the personal opinion of the physician, ADs could lead to a decrease in variability in DFLSTs among physicians. However, there is no discussion between the patient and the physician to explain the wishes expressed in ADs. As a result, the physician may interpret the patient’s wishes differently from what was intended. Our study assessed whether ADs, drawn up by patient after receiving information about DFLSTs and viewing a related video with an intensivist-investigator, would reduce variability in DFLST between intensivists compared to decision-making without ADs. The other aim of the study was to identify the factors associated with DFLSTs or with change in DFLSTs when ADs were available.

## Methods

We conducted a multicenter, prospective, simulation study, in France from September 2017 to March 2018. The study was approved by the local French ethics committee (Comité de Protection des Personnes Sud-Est VI de Clermont-Ferrand (IRB00008526; No. 2016/CE87). A consent form was collected from all participants (patients and intensivists) after they had been informed orally and received a written information form. The study was registered on the ClinicalTrials.gov website under number: NCT03013530 in January 2017 and complied with the guidelines of Strengthening the Reporting of Observational Studies in Epidemiology (STROBE) [18].

### Patients and advance directives

The patients participating in the study were selected from the cohort of consecutive outpatients seen during January–March 2017 for follow-up of chronic disease in the cardiology, pulmonology or nephrology departments of the University Hospital of Clermont-Ferrand. The patient selection criteria were severe stage of chronic cardiac or kidney or respiratory failure associated with comorbid conditions, and life expectancy of less than 5 years according to McCabe score [19], but without acute episode or cognitive impairment on the basis of criteria described by Appelbaum [20]. Of the 23 patients selected, 1 died before the meeting with the intensivist-investigator, 1 was transferred to the palliative care unit, 13 declined and 8 accepted to participate in the study. The characteristics of the 8 patients are shown in Additional file 1. One intensivist-investigator (MS) met each of the eight patients at their home or at a hospital office to provide personalized, clear and full information about DFLSTs and ADs using a video and to suggest that they draw up ADs. The intensivist-investigator had 5 years of ICU practice experience and a master’s diploma in ethics. She was trained in communication skills and in face-to-face conversation about ADs. The video, which lasted 10 min, explained ICU life-sustaining treatment, DFLSTs, and the objectives of ADs as laid out in the guidelines of the French Health Authority. During the meeting, the AD forms of the French Health Authority were given to the patient, who had the opportunity to ask questions about DFLSTs, ADs or end-of-life. The patients were then asked questions to check they had understood the information given. The AD forms comprise 11 pages in which patients can designate a surrogate, express their wishes about life-sustaining treatments in the free-text boxes and 15 pages of guidelines about the writing of wishes, and the use of ADs by physicians in accordance with the French law of 2016 [21], which requires physicians to comply with the patients’ wishes except if the ADs are obviously inappropriate [22]. At the end of the meeting, the patients were invited to draw up their ADs or to take time to discuss with relatives. One patient made a second meeting with the intensivist-investigator to ask further questions. The median time of the meetings was 85.6 [60; 120] min. The ADs were returned within 1 month of the meeting.

### Study procedure

Two clinical scenarios were created by a multidisciplinary team made up of two physicians in palliative care and three intensivists who did not participate in the study. Scenario 1 was followed by six questions and Scenario 2 by four, and both investigated the use of life-sustaining treatment (ICU admission, intubation, renal replacement therapy, vasoactive drugs, tracheotomy) for community-acquired pneumonia with septic shock and for septic shock after gastrointestinal surgery, respectively (see Additional file 2). The possible replies were “yes” or “no” for six questions and “yes” or “partially” or “no” for four questions. The replies were rated 1, 0.5 or 0 if they corresponded to a DFLST, a partial DFLST or no DFLST, respectively. The sum of these ten replies made up a DFLST score ranging from 0 if there was no DFLST to 10 if there was DFLST for all questions. The two scenarios were submitted online to intensivists from 27 French ICUs in 14 university hospitals and in 9 general hospitals. Each intensivist independently and anonymously completed the questionnaires of the two scenarios for each of eight patients with only the patient’s characteristics available (round 1) and then with the same characteristics and the patient’s ADs available (round 2). Round 2 was submitted to intensivists 2 weeks after completion of round 1 (see Additional file 3).

### Statistical analysis

The primary endpoint was variability in DFLSTs between intensivists when ADs were available (round 2) and when they were not (round 1) expressed as the relative standard deviation (RSD; range 0–1; a high rate indicates high inter-intensivist variability). The second endpoint was the identification of factors associated with the DFLSTs or with changes in DFLSTs when ADs were provided. The patient characteristics given to intensivists for the two rounds were age, sex, housing, relatives, medical history, comorbid conditions defined as all diseases or trauma current or previous with the stage or complications reported by the patient or retrieved from medical chart, treatments and dependence in activities of daily living [23]. The recorded characteristics of the intensivists were age, sex, status of intensivist, length of practical experience, speciality of anesthesiology and critical care, religion, interest in ethics, traumatic experience of an end-of-life situation, type of hospital, type of ICU, number of beds and number of intensivists in the ICU, DFLST protocol available in ICU and number of DFLSTs per week.

The number of patients participating in the study was fixed according to the feasibility of answering 20 questions per patient for each round and the case-mix. The sample size of intensivists was fixed to assess how ADs affected the change in variability between intensivists in the DFLSTs. We calculated that at least 120 intensivists evaluating eight patients were necessary to show a 20% relative difference of variability (sample size for a two-sample standard deviations F test), for a two-sided type I error at 5%, a statistical power of 90% and an intra-class correlation coefficient at 0.5, i.e., 960 DFLST scores per round.

All statistical analyses were performed with Stata software (version 15, StataCorp, College Station USA, TX). Continuous data were expressed, according to statistical distribution, as mean and standard-deviation or median and interquartile range. The assumption of normality was studied using the Shapiro–Wilk test. Changes in DFLSTs were compared between rounds 1 and 2 with random-effects models taking into account between and within intensivist variability (intensivists as random-effect). Pitman’s test was used to compare RSD. The agreement on DFLSTs between intensivists (inter-agreement) and the within intensivists agreement (intra-agreement) on DFLSTs between the two rounds for each patient were studied using intra-class correlation coefficient (ICC) estimated by the mixed models mentioned above. ICC was interpreted according to standard recommendations: < 0.2 (negligible agreement), 0.20–0.39 (weak agreement), 0.40–0.59 (moderate agreement), 0.60–0.79 (good agreement) and ≥ 0.8 (excellent agreement).

To determine the characteristics of the ICU physicians associated with DFLSTs in round 1 or with the change in DFLSTs when ADs were provided, random-effects models (i.e., linear mixed models) were carried out with the following parameters as fixed covariates: age, sex, status of intensivist, type of hospital, type of ICU, length of practice and religion. The session effect associated with the two measurement times was studied in the same way. The results are expressed in terms of standardized mean differences and 95% confidence intervals. All analyses were performed with a two-sided type I error of 5%. No missing data were observed.

To evaluate the representativeness of the participating intensivists, a sensitivity analysis was performed to compare their characteristics with those of the non-participating intensivists. Non-participating intensivists were intensivists contacted but who did not reply to all the questions of the two rounds.

A qualitative analysis of ADs was made by a multidisciplinary team composed of two intensivists, one psychologist, one physician in palliative care and two biostatisticians using Alceste software (IMAGE, CNRS, France).

## Results

Of the 170 intensivists contacted, 123 from 27 ICUs completed the two rounds and made up the study population. Their demographic characteristics are given in Table 1. There was no difference between the study population and non-participating intensivists. A total of 19,680 intensivists’ decisions from the two rounds were analyzed.

**Table 1:** Characteristics of intensivists

Variable	Intensivists contacted n = 170	Participating intensivists n = 123	Non-participating intensivists n = 47	*p* value
Male gender, n (%)	113 (63.8)	84 (68.3)	26 (55.3)	0.11
Age (years), mean ± sd	39.9 ± 8.5	40.0 ± 8.5	39.8 ± 8.6	0.88
Status of intensivist, n (%)				
Assistant	37 (21.8)	24 (19.5)	13 (27.7)	
Senior	105 (61.8)	78 (63.4)	27 (57.5)	0.51
Professor	28 (16.4)	21 (17.1)	7 (14.8)	
Length of overall professional experience (years), median [IQR]	6 [3; 15]	7 [3; 15]	6 [3; 15]	0.61
< 5 years, n (%)	60 (35.3)	43 (35.0)	17 (36.2)	
5–15 years, n (%)	73 (42.9)	50 (40.6)	23 (48.9)	0.37
> 15 years, n (%)	37 (21.8)	30 (24.4)	7 (14.9)	
Specialty of anesthesiology and critical care	101 (59.4)	73 (59.3)	28 (59.6)	0.98
Religion, n (%)				
Catholic	66 (38.8)	47 (38.2)	19 (40.4)	
Protestant	5 (2.9)	4 (3.3)	1 (2.1)	
Islam	5 (2.9)	3 (2.4)	2 (4.3)	0.74
Other	10 (5.9)	9 (7.3)	1 (2.1)	
None	84 (49.5)	60 (48.8)	24 (51.1)	
Intensivists with an interest in ethics, n (%)	127 (74.7)	90 (73.2)	37 (78.7)	0.56
Intensivists with a traumatic experience of an EOL situation, n (%)	79 (46.5)	57 (46.3)	22 (46.8)	0.96
ICU in university hospital, n (%)	128 (75.3)	94 (76.4)	34 (72.3)	0.58
Type of ICU, n (%)				
Medical	72 (43.1)	52 (43.0)	20 (43.5)	
Surgery	20 (12.0)	14 (11.6)	6 (13.0)	0.96
Mixed	75 (44.9)	55 (44.4)	20 (43.5)	
Number of beds in ICU, median [IQR]	15 [10;18]	15 [10;18]	13 [10; 18]	0.19
Number of intensivists in ICU, median [IQR]	7 [5;10]	7 [5;10]	7 [5;8]	0.09
DFLST Protocol available in ICU, n (%)	112 (65.9)	77 (62.6)	35 (74.5)	0.14
Number of DFLST performed in ICU, n (%)				
< 1/week	75/158 (47.5)	53/119 (44.5)	22/39 (56.4)	0.20

*DFLST* decision to forgo life-sustaining treatment, *EOL* end-of life, *ICU* intensive care unit, *IQR* interquartile range

### Impact of ADs on the DFLSTs and on the variability in DFLSTs

**Fig. 1:**
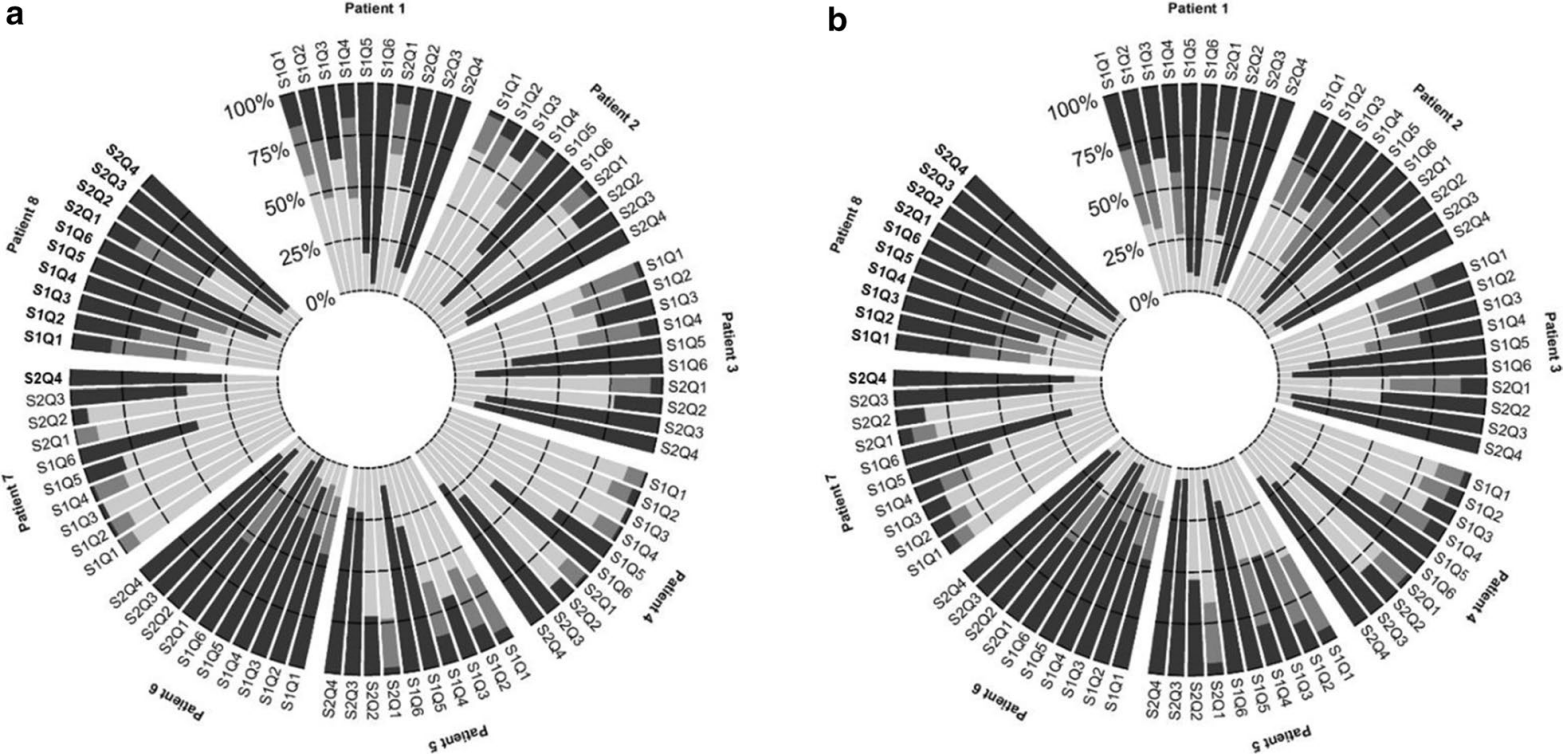
Proportions of DFLSTs in round 1 (**a**) and round 2 (**b**). Legend: Black bars correspond to DFLSTs; Gray bars correspond to partial DFLSTs; White bars correspond to no-DFLSTs. S1: Scenario 1; S2: scenario 2; Q: Question

The proportions of DFLSTs for rounds 1 and 2 are shown in Fig. 1a, b.

The DFLST score was higher with ADs (round 2) than without ADs (round 1) (6.02 95%CI [5.85; 6.19] vs 4.92 95%CI [4.75; 5.10], *p* < 0.001) (Table 2). There was no significant change in inter-intensivists variability when ADs were provided (RSD: 0.56 (round 1) vs 0.46 (round 2), *p* = 0.84) (Table 2). In three patients, the RSD decreased but remained high (Table 2). Inter-intensivists agreement on DFLSTs was moderate (ICC = 0.42) without ADs (round 1) and weak (ICC = 0.28) with ADs (round 2). Intra-intensivists agreements on DFLSTs between the two rounds ranged from weak (ICC = 0.22) to moderate (ICC = 0.56) (Table 2).

**Table 2:** Variability in the DFLST scores (RSD) and the within intensivist agreements (ICC)

	DFLST score						ICC for intensivists
	Median [95% CI]			RSD			
	Round 1	Round 2	*p* value	Round 1	Round 2	*p* value	
Patient #1	5.63 [5.20; 6.06]	7.04 [6.62; 7.47]	< 0.001	0.43	0.34	0.82	0.48
Patient #2	4.18 [3.84; 4.52]	6.31 [5.86; 6.75]	< 0.001	0.46	0.4	0.003	0.22
Patient #3	4.69 [4.29; 5.08]	5.97 [5.53; 6.42]	< 0.001	0.47	0.42	0.18	0.47
Patient #4	2.86 [2.54; 3.18]	3.84 [3.45; 4.22]	< 0.001	0.61	0.56	0.035	0.25
Patient #5	4.70 [4.25; 5.14]	5.81 [5.43; 6.19]	< 0.001	0.53	0.36	0.08	0.38
Patient #6	8.37 [8.02; 8.71]	8.16 [7.76; 8.55]	0.237	0.23	0.27	0.13	0.56
Patient #7	2.42 [2.12; 2.73]	3.72 [3.30; 4.14]	< 0.001	0.71	0.63	< 0.001	0.24
Patient #8	6.54 [6.14; 6.95]	7.32 [6.88; 7.76]	0.001	0.35	0.34	0.44	0.37
All patients	4.92 [4.75; 5.10]	6.02 [5.85; 6.19]	< 0.001	0.56	0.46	0.84	0.56

*CI* confidence interval, *DFLST* decision to forgo life-sustaining treatment, *RSD* relative standard deviation, *ICC*: intra-class correlation coefficient

### Identification of factors associated with the DFLSTs and with the change in DFLSTs when ADs were available

In the univariate analyses of the overall data of the eight patients, no factor associated with the DFLST score in round 1 or with the change in the DFLST score when ADs were available was identified (Table 3).

**Table 3:** Univariate analysis for the identification of factors associated with the DFLSTs and with the change in DFLSTs when advance directives were available

Variable	DFLST score in round 1 Mean ± SD *Correlation coefficient*	*p* value	Change in DFLST score between round 1 and round 2 Mean ± SD *Correlation coefficient*	*p* value
Gender				
Female	5.06 ± 2.72	0.42	1.09 ± 2.48	0.96
Male	4.86 ± 2.80		1.10 ± 2.43	
Age (years)	*0.035*	0.46	*0.027*	0.57
Status of intensivist				
Assistant	4.91 ± 2.91	0.74	0.98 ± 2.48	0.79
Senior	4.98 ± 2.74		1.15 ± 2.42	
Professor	4.74 ± 2.73		1.06 ± 2.53	
Length of overall professional experience				
< 5 years	4.86 ± 2.81	0.23	1.18 ± 2.45	0.42
5–15 years	4.77 ± 2.72		0.94 ± 2.33	
> 15 years	5.27 ± 2.79		1.23 ± 2.63	
Specialty of anesthesiology and critical care				
Yes	4.98 ± 2.76	0.62	1.09 ± 2.46	0.75
No	4.86 ± 2.81		1.15 ± 2.42	
Religion				
Catholic	4.94 ± 2.74	0.74	1.18 ± 2.37	0.78
Protestant	4.75 ± 2.61		0.78 ± 1.90	
Islam	4.06 ± 2.58		0.73 ± 2.97	
Other	4.68 ± 3.06		0.79 ± 2.75	
None	5.00 ± 2.77		1.12 ± 2.46	
Intensivists with an interest in ethics				
Yes	5.01 ± 2.80	0.22	1.14 ± 2.49	0.52
No	4.69 ± 2.70		0.99 ± 2.34	
Intensivists with a traumatic experience of an EOL situation				
Yes	4.79 ± 2.73	0.27	1.27 ± 2.44	0.09
No	5.04 ± 2.80		0.95 ± 2.45	
Hospital type				
General	4.74 ± 2.93	0.37	1.33 ± 2.43	0.18
University	4.98 ± 2.72		1.03 ± 2.45	
Type of ICU				
Medical	4.99 ± 2.81		1.12 ± 2.37	
Surgery	4.81 ± 2.82	0.89	0.56 ± 2.67	0.14
Mixed	4.90 ± 2.73		1.19 ± 2.45	
Number of beds in ICU	*0.062*	0.61	*− 0.10*	0.12
Number of intensivists in ICU	*0.018*	0.87	*− 0.05*	0.28
DFLST Protocol available in ICU				
Yes	5.05 ± 2.80	0.16	1.10 ± 2.37	0.92
No	4.71 ± 2.72		1.11 ± 2.57	
Number of DFLST performed in ICU				
< 1/week	4.70 ± 2.84	0.12	1.15 ± 2.62	0.75
≥ 1/week	5.07 ± 2.69		1.08 ± 2.26	

*DFLST* decision to forgo life-sustaining treatment, *EOL* end-of life, *ICU* intensive care unit, *SD* standard deviation

### Qualitative analysis of ADs

**Fig. 2:**
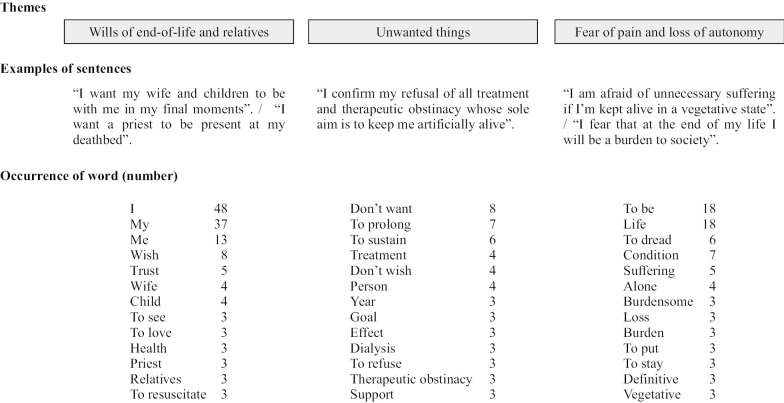
Main themes emerging from the advance directives

A qualitative analysis of ADs was performed on the verbatim texts amounting to 4091 words. Three themes, accounting for 59% of the words, emerged from the ADs: (1) wills of end-of-life and relatives, (2) unwanted things including therapeutic obstinacy and (3) fear of pain and loss of autonomy (Fig. 2). The most frequently occurring words were “life,” “I” and “my.” The word “death” was never stated. The patients expressed themselves in articulate well-structured sentences.

## Discussion

Our results show that when provided with ADs, intensivists made more DFLSTs but this did not alter the great variability between them in their decisions. No factor associated with this variability was identified, suggesting multiple causes that were non-specific to the characteristics of the intensivists. In addition, the qualitative analysis of ADs highlighted the concerns expressed by the patient after they had received full and clear information about DFLST from an intensivist. These results are noteworthy because they show the limitations of the use of ADs.

This study highlights the issue of variability between intensivists in the taking of DFLSTs even when ADs are provided. Great variability has been previously reported in several studies [12, 13, 24–27] and attributed to individual physician characteristics such as religion, culture and geographic regions [7, 8]. The goal of ADs is to enable physicians to comply with the patient’s wishes or request, irrespective of their own personal characteristics [15, 16]. In theory, therefore, ADs should eliminate variability but our findings showed that physicians disagreed about DFLSTs even when ADs were available. There are several possible explanations of this finding. First, the intensivists did not use the ADs to make DFLSTs. However, DFLSTs increased when ADs were available. Second, ADs expressed in free-text boxes led to more possible interpretations of patients’ wishes than ADs drawn up in tick-box form, which includes the use of medical terms for instructions that are easily understood by intensivists and provide clear answers to DFLSTs [28]. In studies assessing the interrater reliability for each section of the POLST form using a binary “yes/no” approach, the Κappa coefficients varied from 0.70 to 1.00 [29]. It is possible that the variability among intensivists is low in the binary “yes/no” approach of DFLSTs wishes. However, the possible replies in our study were “yes,” “no” and sometimes “partial.” Our study differs from these previous reports by the number of questions and the size of the study population, which ensure the robustness of the findings.

A DFLST is a complex but singular process in which the context, the chances of success, the discomfort of treatment, uncertainty regarding prognosis, potential disability and the wishes of the patient are important considerations [30]. ADs with free-text boxes do not allow an exhaustive approach that encompasses in all situations, but give patients the opportunity to express their convictions about the physical or mental impairment [31] that could guide intensivists in their choice of DFLSTs. However, the wording used by the patient can be ambiguous or inappropriate to initiate or to withdraw treatment. The applicability of wishes expressed in ADs results from a match between a hypothetical situation and the medical situation affecting the patient [32]. In our study, the ADs rarely indicated specific wishes about life-sustaining treatments despite information about ICU treatments. This finding could explain the lack of impact of ADs on the high variability between intensivists in the DFLSTs. In this qualitative approach, the influence of relatives or physicians was not assessed. Study of these other areas could improve understanding of the drawing-up, acceptability and usefulness of ADs. The two forms of ADs are complementary and can be associated. After receiving full information during discussion with a healthcare agent, the patient could complete tick-boxes indicating clear DFLSTs that can be implemented whatever the situation and use free-text boxes to express values that could help guide the physician in making DFLSTs in situations not previously considered. The decision about resuscitation status is easily made by the patient and can be registered in a tick-box. In contrast, the initiation of renal replacement therapy is an abstract concept that is discussed according to clinical condition and for which the expression of value is more relevant. The surrogate can add nuances and clarifications to the values relayed in ADs so that the best decisions are made according to the specific condition and prognosis of the patient [33]. A surrogate involved in the drafting of values could limit the influence of the intensivist in the decision-making process using ADs [34]. Alternatively, the physician’s interpretation of values could be assessed in clinical scenarios. Feedback on DFLSTs made by a sample of intensivists in clinical scenarios would allow the patient to modify ADs so that the intensivists’ decisions comply with her or his own and thereby reduce inter-intensivist variability.

Our study has a number of limitations. First, our analyses were performed on the ADs of eight patients. This sample, in agreement with the study feasibility assessment, is not an exhaustive representation of the French population. Nevertheless, the ADs in the study were real documents, which could be used in the decision-making process in clinical practice [35]. Second, to standardize the process, only one intensivist-investigator briefed the patients about DFLSTs and ADs. However, this approach entails the risk of personal influence by the clinician, which can lead to cognitive bias. Third, we do not rule out that the DFLSTs made by intensivists in the scenarios could differ from those taken in everyday practice. However, only a simulation study provides the standardization of situations, which allows assessment of variability between physicians. Our simulation study was time-consuming and unpaid, which could have restricted the participation of some intensivists. The intensivists taking part may have had an interest in ethics and hence were perhaps not representative of the profession as a whole. Fourth, our study design gave no information about family and ICU team discussions or staff opinion. Many DFLSTs are made with non-intensivist physicians, relatives or nurses. Their influence on DFLSTs was not assessed in our study, which focused on the intensivists because they are the main decision-makers for DFLST in the ICU. Fifth, most ADs were collected within 1 month of a single meeting which for some patients could have been too short time to formulate their wishes in full. However, the information about ADs, given by an intensivist-investigator with a video, was of a quality as high as that provided in clinical practice [36] and similar to that used in reference publications [33, 37].

## Conclusions

Our study shows that when ADs were available, intensivists were more likely to make DFLSTs. However, ADs did not reduce high inter-intensivist variability in the decision-making process. The great variability observed show that the intensivist’s preferences had priority. Further research is needed to establish a process that achieves a better matching of the physicians’ decisions with the patient’s wishes.

## Supplementary Information

**Additional file 1.** Characteristics of the 8 patients who wrote advance directives (table).

**Additional file 2.** The two clinical scenarios (text).

**Additional file 3.** Flow chart and timing of the study (Figure).

## Data Availability

The data that support the findings of this study are available from the corresponding author upon reasonable request.

## Abbreviations

ADs: Advance directives
DFLST: Decision to forgo life-sustaining treatment
CI: Confidence intervals
ICC: Intra-class correlation coefficient
ICU: Intensive care unit
POLST: Physician orders for life-sustaining treatment
RSD: Relative standard deviation
