# Supplementary material for: Impact of advance directives on the variability between intensivists in the decisions to forgo life-sustaining treatment
Source: Crit Care. 2020 Dec 2;24:672. doi: 10.1186/s13054-020-03402-7 (PMC7709386; doi:10.1186/s13054-020-03402-7)
Supplement: Supplementary file 1 — Additional file 1. Characteristics of the 8 patients who wrote advance directives (table). [file 13054_2020_3402_MOESM1_ESM.docx]

**Additional file 1**

Characteristics of the 8 patients who wrote advance directives

|  | Age (years) | gender | Housing/  relatives | Comorbid conditions | Number of drugs for chronic treatment | Activities of daily living score* |
| --- | --- | --- | --- | --- | --- | --- |
| Patient 1 | 86 | Male | Lives in a house with his wife | Diabetes, chronic heart failure^‡^ | 8 | 5 |
| Patient 2 | 85 | Female | Lives alone in an apartment | chronic heart failure^‡^, end-stage kidney disease, ankylosing spondylitis | 11 | 6 |
| Patient 3 | 86 | Male | Lives in a house with his wife | chronic obstructive pulmonary disease, lung cancer^‡^, chronic kidney disease | 9 | 6 |
| Patient 4 | 78 | Male | Lives in a house with his wife | chronic obstructive pulmonary disease, chronic heart failure^‡^ | 4 | 6 |
| Patient 5 | 67 | Male | Lives in an apartment with his wife | chronic obstructive pulmonary disease, chronic respiratory failure^‡^ | 6 | 5 |
| Patient 6 | 69 | Male | Lives in a nursing home | chronic obstructive pulmonary disease, chronic respiratory failure^‡^, chronic heart failure^‡^ | 19 | 0.5 |
| Patient 7 | 73 | Male | Lives in an apartment with his wife | chronic heart failure^‡^, end-stage kidney disease, diabetes | 12 | 6 |
| Patient 8 | 84 | Male | Lives in a house with his wife | chronic heart failure^‡^, end-stage kidney disease, chronic obstructive pulmonary disease, bladder cancer^‡^ | 16 | 5.5 |

***:** Activities of daily living score ranging from 0 (total dependence for all activities of daily living) to 6 (total autonomy for all activities of daily living)

‡: Disease related to a life expectancy of less than five years according to McCabe score
