# Supplementary material for: Impact of advance directives on the variability between intensivists in the decisions to forgo life-sustaining treatment
Source: Crit Care. 2020 Dec 2;24:672. doi: 10.1186/s13054-020-03402-7 (PMC7709386; doi:10.1186/s13054-020-03402-7)
Supplement: Supplementary file 2 — Additional file 2. The two clinical scenarios (text). [file 13054_2020_3402_MOESM2_ESM.docx]

**Additional file 2**

The two clinical scenarios

Scenario 1

The patient is admitted to the emergency department for community-acquired pneumonia. The clinical symptoms are: temperature, 38.8°C; heart rate, 120/min; mean arterial pressure, 60mmHg; respiratory rate, 40/min; oxygen saturation of blood, SpO_2_, 75%; dyspnoea and confusion. A chest radiograph shows an extensive air space shadowing throughout 2/3 of the right lung. Antibiotic therapy is prescribed. SpO_2_ is persistently below 85% with oxygen therapy >12L/min.

1) The emergency physician asks you to admit this patient to your intensive care complex. Do you admit this patient to your unit?

a. Yes to the ICU

b. Yes to the high dependency unit* only (no intensive care in case of deterioration)

c. No

2) If only one bed was available in your intensive care complex, would you admit this patient to your unit?

a. Yes to the ICU

b. Yes to the high dependency unit* only (no intensive care in case of deterioration)

c. No

3) The patient was admitted to your ICU and treated with non-invasive ventilation. The SpO_2_ is persistently below 90% with FiO2 >80%. Do you perform endotracheal intubation?

a. Yes

b. No

4) At H6, there is hemodynamic instability resulting from the septic shock. Do you start using vasopressors?

a. Yes

b. Yes, but with a maximum threshold (i.e. 0.5 or 1or 2 microgram/kg/min)

c. No

5) At D8, the patient has been extubated. At D10, pneumonia recurs with acute hypoxemic respiratory failure requiring invasive mechanical ventilation. Do you perform a second endotracheal intubation?

a. Yes

b. No

6) The patient was intubated again. ARDS (acute respiratory distress syndrome) is treated with neuromuscular-blocking drugs and prone position ventilation. At D12, there is persistence of moderate ARDS. Do you make a DFLST?

a. Yes

b. No

Scenario 2

The patient is admitted to the emergency department for intestinal obstruction with pneumoperitoneum. Surgery is performed within the first 6 hours after the admission.

At D5, in the surgical department, the patient has: temperature, 39.2°C; heart rate, 120/min; mean arterial pressure, 55mmHg; oxygen saturation of blood, SpO_2_, 95% and confusion. Hypotension persists after fluid challenge.

1) Do you admit this patient to your unit?

a. Yes to the ICU

b. Yes to the high dependency unit* only (no intensive care in case of deterioration)

c. No

2) At D2 in the ICU, the sepsis is treated with antibiotics and norepinephrine 0.3microgram/kg/min but there is acute hypoxemic decompensated heart failure that persists with non-invasive ventilation. Do you perform endotracheal intubation?

a. Yes

b. No

3) The patient has been intubated. At D7, weaning of positive inotropic agent is performed, but there is acute kidney injury requiring renal replacement therapy. At D18, the patient is again intubated after two attempts at extubation. Do you perform a tracheostomy?

a. Yes

b. No

4) Percutaneous tracheotomy was performed. At D30, the patient is again treated with mechanical ventilation and renal replacement therapy. Do you make a DFLST?

a. Yes

b. No

*: A high dependency unit is a specially staffed and equipped section of an intensive care complex that provides a level of care intermediate between intensive care and general ward care including continuous blood pressure monitoring and non-invasive ventilation or infusion of amine but the invasive mechanical ventilation and the renal replacement therapy are not available.
