# Supplementary figures and images for: Impact of advance directives on the variability between intensivists in the decisions to forgo life-sustaining treatment
Source: Crit Care. 2020 Dec 2;24:672. doi: 10.1186/s13054-020-03402-7 (PMC7709386; doi:10.1186/s13054-020-03402-7)

**Additional file 3**

Flow chart and timing of the study


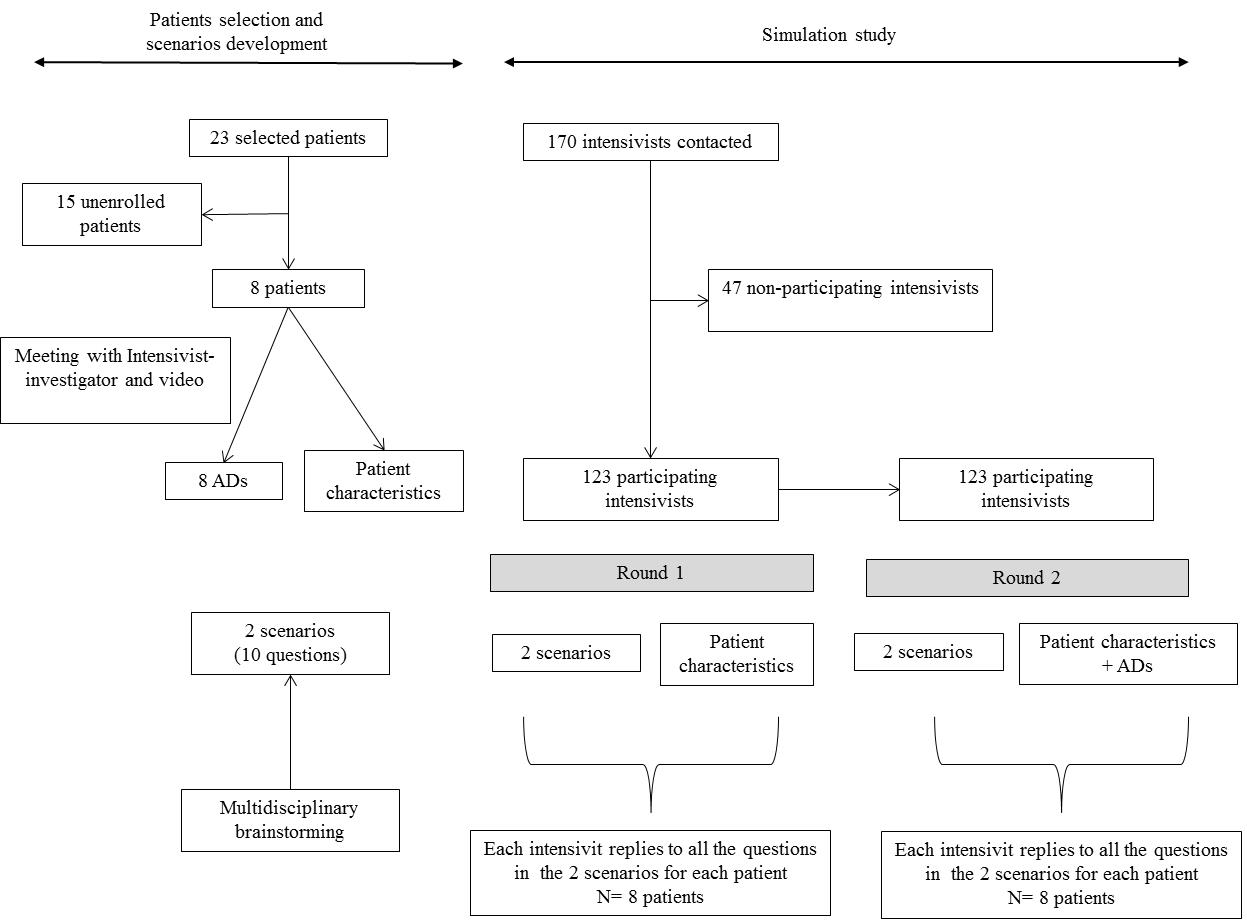

Supplement: Supplementary file 3 — Additional file 3. Flow chart and timing of the study (Figure). [file 13054_2020_3402_MOESM3_ESM.docx]
